# Functional IgG Autoantibodies against Autonomic Nervous System Receptors in Symptomatic Women with Silicone Breast Implants

**DOI:** 10.3390/cells12111510

**Published:** 2023-05-30

**Authors:** Efrosiniia Talalai, Denis Gorobets, Gilad Halpert, Avishai M. Tsur, Harald Heidecke, Yair Levy, Abdulla Watad, Miri Blank, Izhak Michaelevski, Yehuda Shoenfeld, Howard Amital

**Affiliations:** 1 Zabludowicz Center for Autoimmune Diseases, Sheba Medical Center, Tel-Hashomer, Ramat Gan 52621, Israel; ftalalay@gmail.com (E.T.); avishaitsur@gmail.com (A.M.T.); watad.abdulla@gmail.com (A.W.); miri.blank@sheba.health.gov.il (M.B.); yehuda.shoenfeld@sheba.health.gov.il (Y.S.); howard.amital@sheba.health.gov.il (H.A.); 2 Sackler Faculty of Medicine, Tel Aviv University, Tel Aviv 69978, Israel; levy.yair@clalit.org.il; 3 Department of Molecular Biology, Ariel University, Ariel 40700, Israel; denisgo@ariel.ac.il (D.G.); izhakmi@ariel.ac.il (I.M.); 4 Department of Medicine ‘B’, Sheba Medical Center, Tel-Hashomer, Ramat Gan 52621, Israel; 5 Israel Defense Forces, Medical Corps, Ramat Gan 91905, Israel; 6 Department of Military Medicine, Faculty of Medicine, Hebrew University of Jerusalem, Jerusalem 91031, Israel; 7 CellTrend GmbH, 14943 Luckenwalde, Germany; heidecke@celltrend.de; 8 Department of Medicine E, Meir Medical Center, Kfar Saba 44281, Israel; 9 Adelson School of Medicine, Ariel University, Ariel 40700, Israel; 10 Reichman University, Herzelia 46101, Israel

**Keywords:** silicone breast implants, autoantibodies, autonomic nervous system, dysautonomia, G-protein coupled-receptors

## Abstract

The association between the clinical picture of symptomatic women with silicone breast implants (SBI) and dysregulated immunity was in dispute for decades. In the current study, we describe for the first time the functional activity of purified IgG antibodies derived from symptomatic women with SBIs (suffering from subjective/autonomic-related symptoms), both in vitro and in vivo. We found that IgGs, derived from symptomatic women with SBIs, dysregulate inflammatory cytokines (TNFα, IL-6) in activated human peripheral blood mononuclear cells, compared to healthy-women-derived IgGs. Importantly, behavioral studies conducted following intracerebroventricular injection of IgGs derived from symptomatic women with SBIs (who have dysregulated circulating level of IgG autoantibodies directed against autonomic nervous system receptors) into mice brains demonstrated a specific and transient significant increment (about 60%) in the time spent at the center of the open field arena compared with mice injected with IgG from healthy women (without SBIs). This effect was accompanied with a strong trend of reduction of the locomotor activity of the SBI-IgG treated mice, indicating an overall apathic-like behavior. Our study is the first to show the potential pathogenic activity of IgG autoantibodies in symptomatic women with SBIs, emphasizing the importance of these antibodies in SBI-related illness.

## 1. Introduction

Silicone is a foreign material to the human body. Nevertheless, back in the 1960s, silicone was considered as a biologically inert material and has been used since for cosmetic and reconstructive surgical purposes. The safety of silicone implant/injection and its association with immune-related diseases have been debated since its first use [1]. Notably, few years after the first silicone breast augmentation mammoplasty, evidence has been accumulated relating to the occurrence of connective tissue and autoimmune diseases as well as the consequent occurrence of rare cases of breast lymphomas with silicone breast implants (SBI) [2,3]. Throughout the years, many studies have described the prominent association of silicone with dysregulated immunity, both in vitro and in vivo, in animals and humans [4,5,6]. The SBI can cause chronic hyperstimulation of the immune system in genetically predisposed subjects (e.g., HLA gene polymorphism) which can lead to the emergence of clinical autoimmune conditions [7]. Indeed, we previously found the emergence of classical circulating autoantibodies in women with SBIs [8]. Importantly, in a large population-based study, we recently found an elevated risk for developing autoimmune diseases such as Sjogren’s syndrome, systemic sclerosis, and sarcoidosis [9] in women with SBIs. In line with the cited paper above [9], the Food and Drugs Administration (FDA) updated recently its last ‘box warning’ against SBI and stated that patients receiving SBIs have reported a variety of systemic symptoms, including autoimmune diseases [10].

Autoantibodies are one of the key players in the pathophysiology of autoimmune diseases and serve as seminal markers for their diagnosis. Recently, a putative role of IgG autoantibodies has been suggested in the pathophysiology of several autoimmune dysautonomic-related disorders such as chronic fatigue syndrome and postural tachycardia syndrome (POTS) [11]. Our group found a significant change in the level of circulating antibodies directed against G protein-coupled receptors (GPCRs) of the autonomic nervous system in symptomatic women with SBI. These women suffered from various subjective and autonomic-related manifestations such as: cognitive impairment, depression, memory loss, palpitations, and dry eyes and mouth [12]. Notably, these unique autoantibodies (and gene defects in their GPCR target) have been previously described as playing a role in the development of diverse autoimmune diseases [13,14,15,16,17].

A growing body of evidence indicates that a behavioral abnormality may be linked to the effect autoantibodies exert on the central nervous system. Such autoantibody reactivity had been observed not only with cognitive and emotional domains, but also in patients with eating abnormalities, sleep dysfunction, substance addiction, motor dysfunction, etc. [18]

In the current study, we aimed to explore, for the first time, the potential pathogenic effect of circulating IgG antibodies derived from symptomatic women with SBIs, both in vitro and in vivo. These women suffered from subjective symptoms (e.g., cognitive impairment, pain, depression and memory loss) and were found to have dysregulated circulating level of anti-GPCRs of the autonomic nervous system.

## 2. Materials and Methods

### 2.1. Patients

Women with SBIs were recruited from the Zabludowicz Center for Autoimmune Diseases, Sheba Medical Center, Tel-Hashomer, Israel. They arrived at our clinic since they were symptomatic, suffering from clinical manifestations that were suspected to be related to their SBIs. The inclusion criteria of women with SBIs to the study were the following: being symptomatic with a history of breast augmentation procedure (either for cosmetic or reconstruction purposes). The exclusion criteria included having an SBI removed/explanted. Control blood samples from healthy women were provided by the Magen David Adom, Israel’s National Emergency Pre-Hospital Medical and Blood Services Organization.

### 2.2. IRB Approval/Ethical Clearance

The study was approved by the Ethical Committee of the Sheba Medical Center with an approval number of 6619-19-MSC, located at the Sheba Medical Center, Israel. The patients signed a written, informed consent. The animal study received ethical approval from the Ariel University Institutional Animal Care and Use Committee (IACUC), with the assigned approval IL-201-05-20.

### 2.3. IgG Purification from Human Subjects

Total IgG was affinity-purified on a protein g column (Cytiva; Uppsala, Sweden. The sera were loaded on the column and washed with PBS, and bound antibodies were eluted with 0.2 M glycin-HCl pH 2.2, neutralized to pH 7, and dialyzed against PBS. Total IgG concentration was measured using a BCA protein assay kit (Thermo Fisher Scientific; Rockford, IL, USA). IgGs were filtered using a 0.22 µm syringe filter before their use for the in vitro or in vivo experiments.

### 2.4. Human Peripheral Blood Mononuclear Cell Isolation and In Vitro Study

Whole blood was drawn from healthy women into EDTA-containing tubes and processed within two hours of collection. Peripheral blood mononuclear cells (PBMCs) were isolated using lymphoprep™ (STEMCELL Technologies; Bemburg, Germany), according to the manufacturer instructions. PBMCs were cultured in complete RPMI 1640 medium containing heat-inactivated 10% fetal bovine serum (Cytiva; HyClone™ Research Grade; Logan, UT, USA) supplemented with penicillin (100 U/mL; Biological Industries, Beit HaEmek, Israel), streptomycin (100 g/mL; Biological Industries, Beit HaEmek, Israel), and L-glutamine (2 mmol/L; Biological Industries, Israel). The cells were activated (at 37 °C, 5% CO_2_) for 18 h, with or without 2 ng/mL of LPS (Sigma, Rheovot, Israel; *E. coli* O26:B6; Cat: L8274). Total IgG (100µg/mL) derived from healthy women or from symptomatic women with SBI (*n* = 10 for each group; clinical characteristics of blood donors are described in Table 1), were added concomitantly to the LPS-treated cells for a total of 18 h. After 18 h, supernatants were isolated and kept at −80 °C.

### 2.5. Quantification of Cytokine Levels

Human TNFα (DY210-05; R&D Systems), IL-6 (D6050; R&D Systems) and IL-10 (DY217B-05; R&D Systems) ELISA kits were used to quantify cytokine level in supernatants of activated human PBMCs, according to the manufacturer instructions.

### 2.6. Mice and Behavioral Study Design

For the behavioral study, the selection of the ICR outbred strain of mice was based on its stable and consistent genetic background [19]; they have an extensive and wide usage in toxicological [20,21,22] and immunological studies [23,24,25] as well as in behavioral assays [26,27]. Male ICR mice, 12 weeks old, were purchased from Envigo (Israel). The mice (*n* = 10 per group) were subjected to an intracerebroventricular (ICV) injection of a pool of IgGs (*n* = 10, C = 17.2 μg/uL) isolated from the sera of symptomatic women with silicone breast implants (suffering from cognitive impairments, depression, memory loss and widespread pain) vs. a pool of IgGs (*n* = 10, C = 9.16 μg/μL) derived from healthy women (clinical characteristics of blood donors are described in Table 2). The concentration of the antibodies from the symptomatic women was adjusted to the same concentration as those from healthy women by sterile PBSx1 prior to the injections.

Intra-cerebro-ventricular (ICV) injections were carried out utilizing stereotaxic microsurgery, following a modified version of the protocol described by Cetin et al. [28]. Initially, in order to ascertain the precise location of the ventricles under the current experimental conditions, trypan blue microinjections (1 μL) were administered to mice of matching sex, weight, age, and breed prior to the commencement of the experiment. Subsequently, the animals were sacrificed, decapitated, and coronal sections were prepared to visually confirm the ventricular location. The Bregma point was considered as the reference (0 point) for both the anteroposterior (A/P) and mediolateral (M/L) coordinates, while the surface of the skull served as the reference point (0 point) for the dorsoventral (D/V) coordinate. For our mice, the injection coordinates were adjusted as follows: A/P: −0.5; M/L: ±1.60; D/V: −2.50 mm. The injection of the antibody solution was performed unilaterally of a volume of 0.5 μL into each lateral ventricle, resulting in a total antibody dosage of 9.16 μg/μL × 0.5 μL × 2 = 9.16 μg. Glass capillary stereotaxic syringes (Hamilton, NY, USA) were used for the injections, with a flow rate of 0.1 μL/min. Following the surgery, wound closure was achieved using a tissue adhesive wound-sealant (RiverPoint, Tanner, WA, USA). The surgeries were conducted in the early morning to allow the mice ample time to recover before the subsequent day’s habituation period.

**Figure 1:**
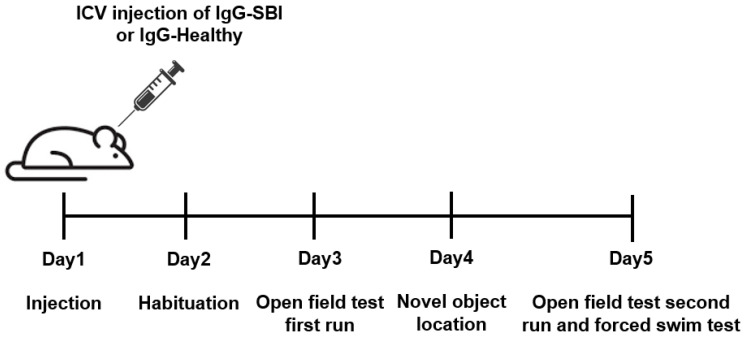
Experimental timeline. A single intraventricular (ICV) injection of a pool of IgGs isolated from the sera of symptomatic women with silicone breast implants (*n* = 10; IgG-SBI) or from healthy women (*n* = 10; IgG-healthy) was administrated into ICR male mice (*n* = 10 per each group) on Day 1. Naïve untreated mice were used as controls. Various behavioral studies were conducted in the following four days.

Naïve mice (*n* = 10) which were not subjected to any treatment were used as control animals. Due to unknown half-life of the antibodies in the cerebrospinal fluid (CSF) and brain tissue, to minimize false negative outcomes, behavioral paradigms were commenced two days after the injection. The experimental design (described in Figure 1) included the testing of 10 mice from each group, which were split into two sets containing 5 mice in each experimental group. All procedures were conducted using ICV injection and followed by subsequent behavioral paradigms which were accomplished in 5 days: day 1—injection and recovery, day 2—habituation, day 3—open field test first run, day 4—novel-object location (short-term memory version), day 5—open field second run and forced swim test.

### 2.7. Open Field Test (OFT)

The OFT test [29,30,31,32] was used to estimate a spontaneous locomotor activity and anxiety behavior, and was performed in a standardized arena of 40 × 40 cm made from inert plastic (Maze Engineers, Skokie, IL, USA). For a better habituation, all the animals were placed in the experimental room one hour prior to the test. The apparatus was cleaned with 70% ethanol between sessions. Each mouse was placed individually for 6–10 min in the square black plastic chamber. Mice movements were recorded using a camera, movement tracking and automatic scoring were performed using EthoVision XT 11.5 (Noldus, Wageningen, The Netherlands) software. All variables extracted from the OF test, including % of time at the center/corner/margin, walking velocity, and distances for all three experimental groups, were subjected to factor analysis utilizing a principal factor extraction algorithm.

### 2.8. Novel-Object Location (NOL)

A novel-object location (NOL) paradigm was used to evaluate spontaneous memory [33,34]. Tests were performed using an opaque arena of 40 cm × 40 cm × 40 cm and were divided into 3 sessions. During the training session (10 min), animals were placed in the arena center and habituated to 2 identical objects placed at fixed locations in the arena. To test short-term memory (within 30 min after the training session), 1 of 2 objects were placed in another location in the arena, which allows us to estimate the ability of the animal to recognize the new location of the object [35]. The time spent within 2 cm of each object was recorded during a 5 min period. For a better habituation, all animals were placed in the experimental room one hour prior to the test. Between subjects, the apparatus was washed with 70% ethanol.

The percentage of time spent exploring the novel location of the object relative to the total time spent exploring both objects was used to evaluate preference index (PI). It is represented by the formula: [PI = NL/(NL + OL) × 100%] where PI—a preference index; NL—time spent investigating the novel location of moved object; and OL—time spent investigating an object which was not moved and stood in its old location. A value above 50% means greater investigation of the novel location of the object.

### 2.9. Forced Swim Test (FST)

The FST [29,36] is an acute environmental stressor, which measures behavioral despair and has been described as a primary screening test for antidepressants’ effect in mice [37]. During the test, the mice were placed individually into a glass cylinder (30 height × 10 cm diameter) filled with water up to 25 cm height (25 ± 2 °C) for 6 min and the immobility time was recorded [38]. A non-swim time was measured as the time the mice spent immobile and aims to reflect the behavioral and the despair characteristics of depressed individuals. Immediately after the test, the mice were then dried with paper towels, warmed under a lamp for 10 min, and returned to their home cages. The animals which failed to stay afloat were removed immediately.

### 2.10. Quantification of Circulating Auto-Antibody Levels

Whole blood samples from each SBI and healthy control subject were allowed to clot at room temperature, and then centrifuged at 2000× *g* for 15 min in a refrigerated centrifuge. The serum was purified and stored at −35 °C. The anti-adrenergic receptors (α1, α2, β1, β2), anti-muscarinic receptors (M1–M5), anti-endothelin receptor type A, and anti-angiotensin II type 1 receptor IgG autoantibodies were measured in serum samples as described previously [12,17,39,40] using a sandwich ELISA kit (CellTrend GmbH Luckenwalde, Germany). The microtiter 96-well polystyrene plates were coated with the GPCRs. To maintain the conformational epitopes of the receptor, 1 mM calcium chloride was added to every buffer. Duplicate samples of a 1:100 serum dilution were incubated at 4 °C for 2 h. After washing steps, plates were incubated for 60 min with a 1:20,000 dilution of horseradish-peroxidase–labeled goat anti-human IgG used for detection. In order to obtain a standard curve, the plates were incubated with the test serum from an anti-GPCR-autoantibody-positive index patient. The ELISAs were validated according to the FDA’s “Guidance for industry: Bioanalytical method validation”. The optimal cut-off level for each anti-GPCR autoantibody test was analyzed using the receiver operating characteristic (ROC) analysis as described previously [41].

### 2.11. Statistical Analysis

Collected data were subjected to descriptive statistical analysis to evaluate the data quality and distribution parameters. Multimodal normal distribution was assessed with the Gaussian multicomponent fit equation. Data were subjected to outlier analysis using Grubb’s test. Kolmogorov–Smirnov and Shapiro–Wilk tests were used for data normality assessment. Univariate analysis on more than 2 groups was conducted using One-way ANOVA with a subsequent Holm–Sidak post hoc analysis for pairwise comparisons. A Kruskal–Wallis one-way analysis of variance with a Tukey post hoc method was used for data not distributed normally. No data were subjected to log or square-root transformation. Data were presented as mean and standard error of mean (SEM) or as median and interquartile range using box-and-whisker plots. Statistical test outcomes were presented by the *p* and t values for the pairwise comparisons with post hoc analysis, *p* and F values for One-way ANOVA, and *p* and q values for KW one-way ANOVA with Tukey post hoc analysis.

Bootstrapping analysis was conducted for setting the test group size to 10,000 samples, with subsequent statistical analysis to evaluate the distributions’ descriptive statistics and the validity of the observed trends.

Multivariate exploratory factor analysis was used for multivariable data, linear decomposition and potential factor loading evaluation. A principal component factor extraction method was used. Any factor with an eigenvalue less than 1 was discarded. Enriched factor loadings were subjected to varimax orthogonal rotation. Data was also assessed for hidden dependence using an oblique rotation method. Higher than a 70% correlation was considered as significant for factor loading on variables. Data was analyzed using GraphPad Prism software Version 7.0 (GraphPad Software, Inc. La Jolla, CA, USA).

## 3. Results

### 3.1. IgG Derived from Symptomatic Women with Silicone Breast Implants Dysregulates LPS-Induced Cytokine Secretion in Activated Human PBMCs

**Figure 2:**
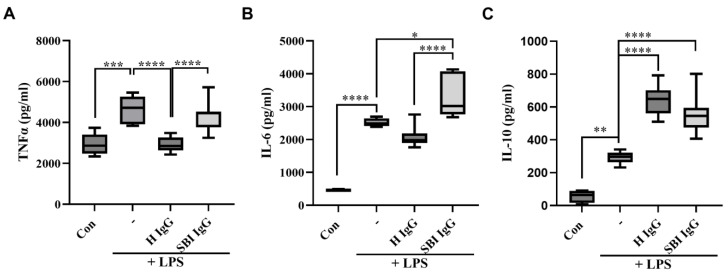
IgGs derived from women with silicone breast implants dysregulate LPS-induced cytokine secretion in activated human PBMCs. Total IgGs were isolated from healthy women (*n* = 10) or from symptomatic women with silicone breast implants (*n* = 10). Human PBMCs were isolated from healthy woman, seeded (500,000 cells/0.5 mL/well) and activated using LPS (2 ng/mL) for 18 h. Total IgGs (100 µg/mL) from each group were added concomitantly with LPS for 18 h. (**A**) TNFα, (**B**) IL—6 and (**C**) IL—10 cytokine levels were measured in the supernatants, 18 h after activation, by ELISA assay. Box-and-whisker plots showing medians and interquartile ranges of each cytokine level. The dark line within each box represents the median while top and bottom borders of the box represent the third and first quartiles. * *p* < 0.05, ** *p* < 0.01, *** *p* < 0.001, **** *p* < 0.0001. SBI—silicone breast implant, LPS—lipopolysaccharides, PBMCs—peripheral blood mononuclear cells, H—healthy.

We first aimed to explore the potential in vitro activity of IgGs derived from symptomatic women with SBIs (*n* = 10, suffering from subjective/autonomic-related symptoms; Table 1) compared to IgGs derived from healthy women (*n* = 10; Table 1), in human primary immune cells. Peripheral blood mononuclear cells (PBMCs) isolated from a healthy woman were cultured with LPS (2 ng/mL) and incubated simultaneously with either healthy-women-derived or women-with-SBI-derived total IgGs (100 µg/mL) for 18 h, after which the levels of TNFα, IL-10 and IL-6 were quantified in culture supernatants. It has been previously shown that polyclonal IgGs from healthy individuals inhibit LPS-induced TNFα and enhance IL-10 production and its secretion in human monocytes [42]. Consistent with these data, IgGs derived from healthy women significantly reduced LPS-induced TNFα secretion by PBMCs (*p* < 0.001; Figure 2A). In contrast, IgGs derived from symptomatic women with SBIs had no such inhibitory effect. Furthermore, while healthy-IgGs show a trend in the reduction of IL-6 secretion by PBMCs, SBI-IgG significantly upregulated its secretion (ns and *p* < 0.05, respectively; Figure 2B). With regard to IL-10, both groups significantly increased IL-10 secretion, though this effect was found to be less pronounced for IgGs derived from women with SBIs compared to healthy-IgGs (*p* < 0.01 vs. *p* < 0.001, respectively; Figure 2C).

### 3.2. Passive Transfer of IgGs Derived from Symptomatic Women with SBI, into Mouse Brain, Affects Anxiety-like Behavior and Locomotor Activity

**Figure 3:**
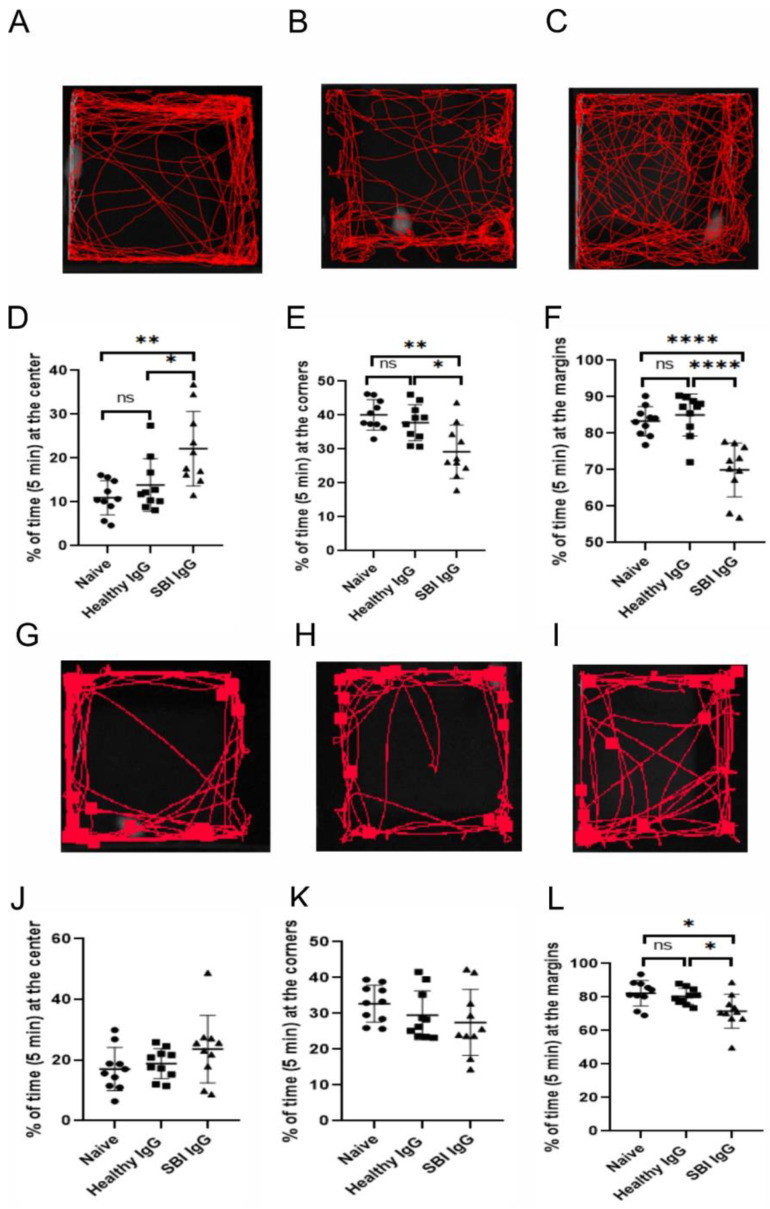
Passive transfer of IgGs, derived from symptomatic women with SBIs, into mice brain effects anxiety-like behavior. Open field test conducted on the third and fifth experimental day (two days and four days after injection—(**A**–**F**) and (**G**–**L**), respectively). (**A**–**C**) representative mouse trajectories for naïve (**A**), healthy-IgG- (**B**) and SBI-IgG-injected (**C**) ICR mice. (**D**,**E**) Parameters derived for open field test performance: relative time (in %) spent at the center (**D**), corners (**E**) and margins (**F**) in ratio to the total time in the arena (5 min). (**G**–**I**) as (**A**–**C**) and (**J**–**L**) as (**D**–**F**), respectively. Ten mice in each group were checked in two experimental sets (5 mice in each group per each set). Data presented as an individual point spread, mean and standard error of mean (bars). * *p* < 0.05, ** *p* < 0.01, **** *p<* 0.0001, ns—non-significant. Circles—Naïve group, squares—Healthy IgG group, triangles—SBI IgG group.

A pool of IgG antibodies isolated from the sera of symptomatic women with silicone breast implants (*n* = 10, suffering from cognitive impairment, depression, memory loss and widespread pain; Table 2) vs. IgGs derived from healthy women (*n* = 10; Table 2), were injected (intracerebroventricular (ICV) administrations) into the brain of ICR mice (Figure 1). Notably, the circulating titer of anti-β1 adrenergic receptor and anti-angiotensin II type 1 autoantibodies in these 10 symptomatic women with SBIs were significantly reduced vs. the 10 healthy women (*p* < 0.0001 and *p* < 0.001, respectively; Table 2). The changes in the titer of these antibodies is consistent with our recent observation regarding the importance of anti-β1-adrenergic-receptor autoantibodies in SBI-related illness and their significant correlation with autonomic-related manifestations reported by women with SBIs, such as depression [12]. The assessment of the animals’ behavior in the open field paradigm, two days after the injection (Day 3), revealed a strong preference of SBI-IgG injected mice for the field center crossing vs. the clear lateralization of naïve and healthy-IgG-injected ones (Figure 3A–C). Visual observations of the animal trajectories were further supported by a significant increase of time spent at the center in SBI-IgG-injected mice vs. the other two groups (*p* < 0.01, F_2,29_ = 8.195, one-way ANOVA with a Holm–Sidak post hoc, *p* < 0.01 SBI-IgG vs. Naïve (t = 3.902) and *p* < 0.05 SBI-IgG vs. healthy-IgG (t = 2.884); Figure 3D), as well as a corresponding marked decrease in the time spent at the corners (*p* < 0.01, F_2,29_ = 8.933, one-way ANOVA, with a Holm–Sidak post hoc, *p* < 0.01 SBI-IgG vs. Naïve (t = 4.012) and *p* < 0.05 SBI-IgG vs. healthy-IgG (t = 3.158); Figure 3E) and at the margins (*p* < 001, H_2,29_ = 17.21, Kruskal–Wallis One-way analysis of variance with ranks, with a Tukey post hoc addition, *p* < 0.001 SBI-IgG vs. Naïve (q = 5.496) and *p* < 0.001 SBI-IgG vs. healthy-IgG (q = 4.526); Figure 3F). Such dramatic preference for the arena center crossing vs. lateralization indicates that the SBI-IgG injection induced anxiolytic effects in the treated animals. Validity of the open field analysis was further supported by conducting the behavioral paradigm four days after the injection (Day 5), while a complete disappearance of the SBI-IgG effect was observed along with no significant effect on the animals’ preference for the center, though a slight trend was still identifiable (Figure 3G–L). The obtained results were further supported by analysis of the cumulative immobility time, which revealed that two and four days after the injection (Day 3 and Day 5), SBI-IgG-injected mice were immobile in the center of arena significantly longer than the other groups (*p* < 0.001, F2,27 = 17.48). Moreover, four days after the injections (Day 5), the immobility state of these mice was preserved (no significant difference in the time effect: *p* = 0.09, F1,27 = 2.92; two-way ANOVA; Supplementary Figure S2A,B). SBI-IgG-treated mice also revealed a further distance traveled within the center of the arena (*p* < 0.05, F2,27 = 4.97; two-way ANOVA), despite the total distance travelled within the whole arena remaining almost equal between the groups (Supplementary Figure S2A,B).

**Figure 4:**
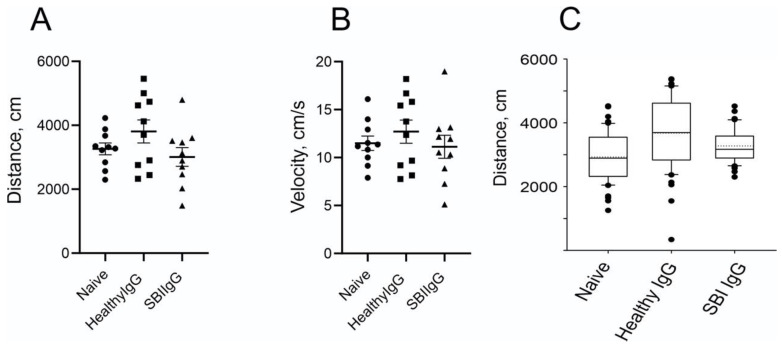
Passive transfer of IgGs, derived from symptomatic women with SBIs, into mice brains causes a reduction in locomotor activity. Locomotory activity parameters derived from open field tests as in Figure 3. (**A**) Summarized distance traveled by the tested animals as derived from trajectory files. (**B**) Average velocity of the animal movement calculated for the trajectories during the test elapsed time. Data presentation as in Figure 3D,E. (**C**). Summarized box-and-whisker plots of bootstrapped data derived from the simulation based on distribution of traveled distances as presented on Supplementary Figure S1. Circles—Naïve group, squares—Healthy IgG group, triangles—SBI IgG group.

Further analysis of the locomotory activity of the animals in the arena (two days after injection; day 3) did not reveal significant differences in the distance moved or running velocity; however, closer inspection of the data shows a trend of moved-distance and velocity reduction in SBI-IgG-injected mice that may indicate more apathetic behavior vs. healthy-IgG-injected ones (Figure 4A,B). The effect on locomotor activity four days after the injection (Day 5) was abolished.

Interestingly, the implementation of bootstrapping analysis to simulate the similar data behavior on the larger population maintained and even enhanced the trend and heightened the multimodal behavior of the distribution (Figure 4C; Supplementary Figure S1A–C). Hence, further goal-directed behavioral analysis is necessary to solidify this notion.

**Figure 5:**
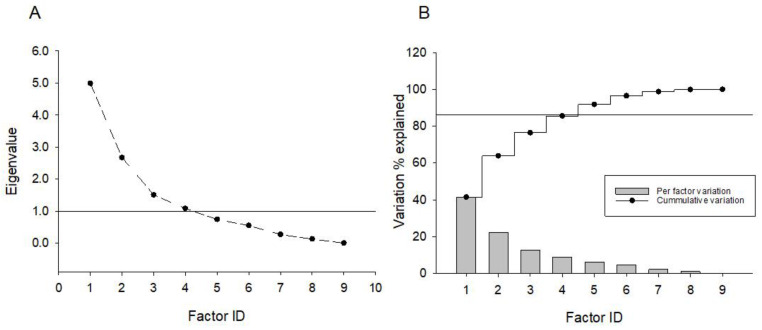
Exploratory factor analysis applied to variables generated from open field paradigm data (see Supplementary Table S1). Principal component-based factor extraction method with subsequent varimax orthogonal rotation was used to derive factor loadings on variables. (**A**) Number of factors derived from the screen plot analysis; an eigenvalue of 1 has been used as a principal factor definition threshold. (**B**) Percentage of variation explained by a specific factor (bar graph) and cumulative variation explanation percentage (splay lines). No secondary factors were detected upon oblique rotation application.

The effect of SBI-IgG injection on mouse locomotory activity was further validated by applying factor analysis to the whole open field paradigm data. Using a principal component factor extraction method with a varimax orthogonal rotation, four orthogonal factors were identified (Figure 5A) totally explaining 85.5% of the data variation (Figure 5B). Factor 1 exhibited the highest positive correlation with margin-oriented healthy-IgG-injected mice and, similarly, the highest negative correlation was found between the healthy-IgG-injected mice and center orientation, while factor 2 showed a similar correlation pattern with SBI-IgG-injected mice. Orthogonality of the factors further supports the statistically significant data shown in Figure 3. Factors 3 and 4 demonstrated the highest correlation with SGI-IgG mice and healthy-IgG-injected mice velocity, respectively (Figure 4). Orthogonality of the factors validates a biological significance of the data shown in Figure 4, indicating a marked effect of SBI-IgG injection on mouse locomotor activity.

A novel-object-location paradigm was performed three days after injection (Day4), while the day of the injection (Day 1) and the day after (Day 2) were used for habituation reasons (Figure 1). The choice of the novel-object location paradigm was also related to the ability to use the same arena as for the open field test. During the training phase, all the animals demonstrated similar preferences towards both locations of the objects (Supplementary Figure S3B,D,E), while the positioning of the explored object to a novel location did not lead to any prominent preference change in the mice of any group, as expressed by the non-significant differences in the times spent near the old and new locations of the object, and the novel location preference index assessment disregarding the method of measuring (Supplementary Figure S3D,E). Notably, a visual inspection of animal trajectories still revealed differences upon the objects movement to a novel location in SBI-IgG-injected mice, though a similar impact was observed in healthy-IgG-injected ones. Based on these findings, no cognitive impact on short-term recognition memory was identified. However, the absence of an effect may be related to the very short half-life of the antibodies in brain tissue, and CSF as could be seen on the fifth experimental day (four days after IgG injection)—indicating no effect in the open field paradigm (Figure 3).

The forced swim test is considered the golden standard test for depressive-like behavior. Based on observations from our recent publication showing that approximately 40% of symptomatic women with SBIs suffer from depression [12], an increase in immobility time in SBI-IgG-injected mice was anticipated. However, no significant differences in immobility times between groups were observed (Supplementary Figure S3A). Notably, the FST paradigm was performed after the second OFT, which was also found to be similar between the tested groups, indicating the disappearance of SBI IgG effect by this point, potentially due to its elimination from CSF and brain tissue.

## 4. Discussion

The potential pathogenic functional effect of IgG antibodies in the blood of women with silicone breast illness has not been explored. Our study is the first to examine the functional activity of IgG antibodies derived from symptomatic women with SBIs, both in vitro and in vivo.

The so called ‘natural’ protective autoantibodies were described a few decades ago and have been shown to have physiological roles in our body: the inhibition of NK cell activity, the inhibition of IFNγ activity by binding to the cell surface, the neutralization of pathogenic autoantibodies by anti-idiotypic antibodies, etc. Our in vitro results showed that healthy-women-derived IgG antibodies significantly reduced TNFα production and significantly increased IL-10 in LPS-induced activated human PBMCs (Figure 2). These results are in agreement with the previously described anti-inflammatory effect of intravenous immunoglobulins (IVIG), a pool of 1000–10,000 healthy IgGs, both in vitro on activated murine and human monocytes/macrophages [43,44], and in vivo in experimental autoimmune diseases [45]. Importantly, we found that SBI-IgG preparations failed to reduce TNFα production while they significantly increased IL-6 in LPS-induced activated human PBMCs, emphasizing the dysregulated effect of these antibodies on the production of inflammatory mediators by immune cells.

Importantly, we found that the passive transfer of IgGs derived from symptomatic women with SBI into mice brains effects anxiety-like behavior and affects locomotor activity and, overall, induces animal apathetic behavior (Figure 3 and Figure 4). Notably, these symptomatic women with SBIs were found to have dysregulated titers of circulating anti-GPCR autoantibodies directed against autonomic nervous system receptors, and suffered subjective manifestations such as cognitive impairment, depression and widespread pain (Table 2). These findings suggest an important role of these IgG autoantibodies in the development of SBI-related subjective and autonomic manifestations and strengthen the autoimmune basis for this disorder [9]. As some of the autoantibodies are present in blood as part of immune complexes, it is possible that after isolating IgG antibodies using a protein g column technique, these immune complexes may break down. Therefore, the functional activity, both in vitro and in vivo, of IgG antibodies derived from symptomatic women with SBIs in our study might be different than the origin of antibodies present in the blood.

It is worth mentioning that dysregulated autoantibodies targeting neurotransmitter receptors or other neuronal antigens have been linked to major depressive disorders (MDD) [46,47]. For example, numerous reports had linked anti-opioid receptor (OPRM1) IgG autoantibodies [48], antibodies to 5HT-1A or 5-hydroxytryptamine receptor 1A (HTR-1A), DRD2, and antibodies directed against the GPCR muscarinic cholinergic receptor 1 (CHRM1), to MDD [49]. Not surprisingly, the comorbidity of MDD and autoimmune diseases has been reported. For example, our group showed that ICV injection of antibodies directed against ribosomal-*p* (isolated from SLE patients) induced depression-like behavior in mice [36,50]. We also showed that a passive transfer of anti-TRIB2 IgG autoantibodies, isolated from patients with narcolepsy, induced neural loss and sleep attacks in mice [29]. Lapteva et al. found that antibodies raised against different subunits of NMDARs which were also associated with depression and cognitive impairment in systemic lupus erythematosus [51,52].

Recently, the importance of IgG antibodies in the pathophysiology of other suspected autoimmune dysautonomic-related disorders has been suggested. For example, fibromyalgia-related symptoms were successfully transferred from patients into mice, using passive transfer of IgG antibodies [53]. Moreover, Carmen et al. [54] found that immunoadsorption of IgG autoantibodies against β2 adrenergic receptors may lead to a clinical improvement of chronic-fatigue-syndrome patients. The same group later showed the functional activity of these CFS-derived IgG autoantibodies in vitro [42]. Those studies clearly show an autoimmune basis for these disorders, suggesting that treatment aimed at reducing the levels of IgG autoantibodies directed against GPCRs of the autonomic nervous system might be an optional mode of therapy. Notably, symptomatic women with SBI shared enigmatic, subjective and autonomic-related manifestations as in CFS and fibromyalgia syndrome, such as severe fatigue, widespread pain, cognitive impairment and depression [11,12]. However, we urge for the increased awareness of this and for the possibility to screen for potential dysregulated titers of circulating anti-GPCR IgG antibodies in patients with suspected autoimmune dysautonomia.

There are few limitations to our study. The behavioral tests used in our preliminary study involved local ICV injections of IgG antibodies derived from a pool of only 10 samples per group. Future experiments should consider the use of peripheral intravenous injections of IgG antibodies isolated from the serum of each subject separately (from either patients or a healthy group), which will probably strengthen the potential pathogenic effect of these antibodies and their brain penetration as well. Furthermore, the use of specific IgG antibodies (e.g., specific anti-GPCRs of the autonomic nervous system such as anti-β1 adrenergic receptor) instead of total IgGs in both in vitro and in vivo experiments will prove a direct effect of the autonomic nervous system in SBI-related illness.

Overall, our study strengthens the potential in vivo pathogenic activity of IgG antibodies derived from symptomatic women with SBIs. Moreover, our study emphasizes the importance of these antibodies in SBI-related illness and other suspected autoimmune dysautonomic-related disorders [55].

## Figures and Tables

**Table 1:** Clinical characteristics of blood donors; in vitro study.

Patient Characteristics	Women with SBI *n* = 10	Healthy Women *n* = 10
Mean age ± SEM (y)	51.1 ± 3.9	39.5 ± 4.9
Mean time from implantation to symptom onset ± SEM (y)	9.3 ± 2.8	
Cosmetic indication for SBI	7 (70%)	
**Subjective/Autonomic-related symptoms**		
Fatigue	8 (80%)	
Widespread pain	7 (70%)	
Depression	4 (40%)	
Cognitive impairment	5 (50%)	
Memory impairment	6 (60%)	
Palpitations	3 (30%)	
Dry mouth	3 (30%)	
Sleep disturbances	7 (70%)	
**Medical History**		
Autoimmune thyroid disease	4 (40%)	
Inflammatory bowel disease	0 (0%)	
Endometriosis	0 (0%)	
**Familial history for autoimmune disease**	4 (40%)	
**Drug Use**		
Selective serotonin reuptake inhibitors (Cipralex)	2 (20%)	
Stimulants (Ritalin)	0 (0%)	
TNF blockers (Humira)	0 (0%)	
Immunosuppressants (Azathioprine)	0 (0%)	

**Table 2:** Clinical characteristics of blood donors; in vivo studies.

Patient Characteristics		Women with SBI *n* = 10	Healthy Women *n* = 10
**Mean age ± SEM (y)**		**37.7 ± 1.7**	**37.8 ± 4.1**
Mean time from implantation to symptom onset ± SEM (y)		10.6 ± 1.2	
Cosmetic indication for SBI		10 (100%)	
**Subjective/Autonomic-related symptoms**			
Fatigue		10 (100%)	
Widespread pain		10 (100%)	
Depression		10 (100%)	
Cognitive impairment		10 (100%)	
Memory impairment		10 (100%)	
Palpitations		9 (90%)	
Dry mouth		8 (80%)	
Sleep disturbances		8 (80%)	
**Medical History**			
Autoimmune thyroid disease		1 (10%)	
Inflammatory bowel disease		3 (30%)	
Endometriosis		1 (10%)	
**Familial history for autoimmune disease**		5 (50%)	
**Drug Use**			
Selective serotonin reuptake inhibitors (Cipralex)		1 (10%)	
Stimulants (Ritalin)		1 (10%)	
TNF blockers (Humira)		1 (10%)	
Immunosuppressants (Azathioprine)		1 (10%)	
**Sera autoantibody** **median levels** **(Q25, Q75; units/mL)**	**Healthy women** ***n* = 10**	**Women with SBI** ***n* = 10**	***p*-Value**
Anti-β1 adrenergic receptor	47.8 (22.8, 54.6)	8.4 (7.4, 10.2)	0.000 ****
Anti-angiotensin II type 1 receptor	27.6 (13.3, 43.9)	10.4 (9.9, 11.9)	0.007 **
Anti-endothelin receptor type A	14.7 (10.8, 17.9)	9.0 (7.8, 10.1)	0.087

*p*-values by unpaired two-samples *t*-test. ** *p* ≤ 0.01, **** *p* ≤ 0.0001.

## Data Availability

The data presented in this study are available on request from the corresponding author. The data are not publicly available due to privacy and ethical concern.

## Supplementary Materials

The following supporting information can be downloaded at: https://www.mdpi.com/article/10.3390/cells12111510/s1, Figure S1: Bootstrapped data derived from simulation based on distribution of traveled distances. Figure S2: Immobility time and moved-distance analysis in open field test. Figure S3: Forced swim test and short-term version of novel-object location paradigm. Table S1: Factor loadings derived from varimax orthogonal rotation.

## Abbreviations

SBI: silicone breast implants; GPCR, G-protein coupled receptor; CSF, cerebrospinal fluid; OFT, open field test; NOL, novel-object location; FST, forced swim test; ICV, intracerebroventricular; MDD, major depressive disorders.
