# Supplementary material for: Functional IgG Autoantibodies against Autonomic Nervous System Receptors in Symptomatic Women with Silicone Breast Implants
Source: Cells. 2023 May 30;12(11):1510. doi: 10.3390/cells12111510 (PMC10252975; doi:10.3390/cells12111510)
Supplement: Supplementary file 1 [file cells-12-01510-s001.zip › cells-2381707-supplementary.pdf]

Supplementary Table S1: Factor loadings derived from varimax orthogonal rotation. Bold fonts denote factors with maximal loadings on variables. Absolute value of 0.7 denotes significant correlation between the factors and variables.

Var/Fact	Factor 1	Factor 2	Factor 3	Factor 4
n-vel	-0.31258	-0.58612	0.129564	0.043169
**h-vel**	0.08962	0.068127	0.068364	**0.972943**
**s-vel**	-0.24837	-0.25202	**0.835511**	0.284956
n-center	-0.89255	-0.19604	0.03754	0.153862
**h-center**	**-0.94213**	0.150922	0.15712	-0.05103
**s-center**	0.067447	**-0.90292**	-0.28188	-0.07561
n-corner	0.807638	0.336838	0.153048	0.394285
h-corner	0.603094	0.221748	-0.50304	0.351449
s-corner	0.03073	0.415601	0.754129	-0.08403
n-margin	0.737266	0.569087	-0.14574	0.107134
**h-margin**	**0.942126**	-0.15092	-0.15712	0.05103
**s-margin**	-0.04096	**0.948798**	-0.04814	0.097211

Supplementary figures:

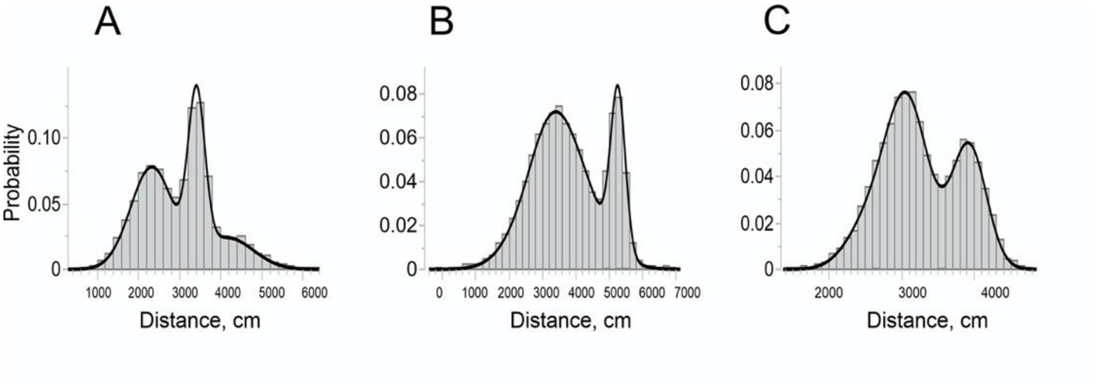

Supplementary Figure S1: Bootstrapped data derived from simulation based on distribution of traveled distances. (**A**)—Naïve, (**B**)—healthy IgG, (**C**)—SBI IgG injected. Simulation was conducted for 10,000 points in each group. Obtained data has been sampled and the histogram was fitted using 3-mixed component gaussian function (solid black line depicting fit).

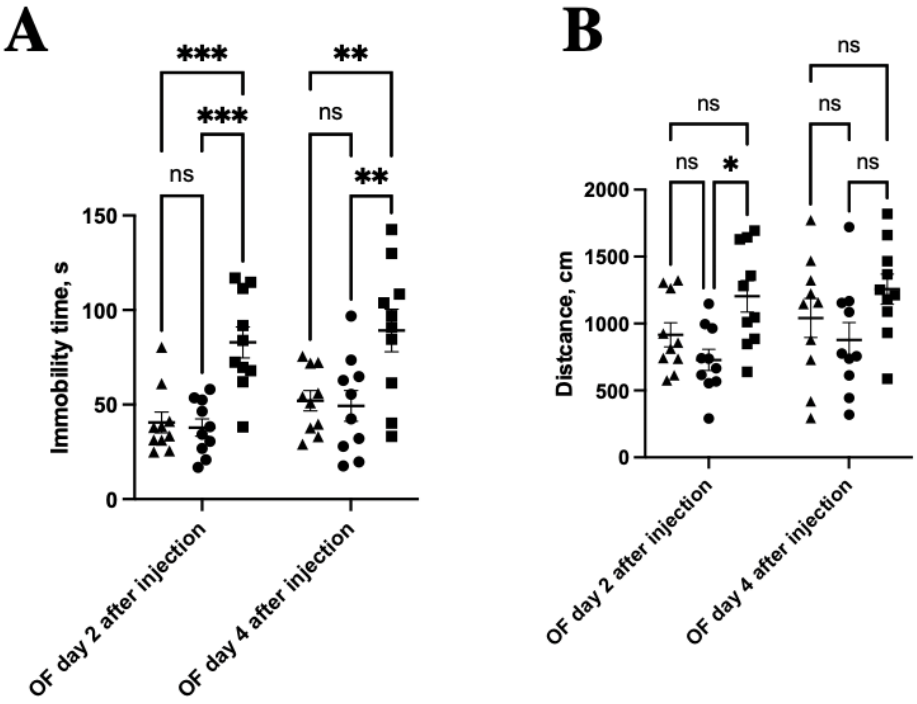

Supplementary Figure S2: Immobility time and moved distance analysis in open field test. (**A**). Immobility time was analyzed as immobile state cumulative time, counted as time where velocity of animal was under 20% of the threshold of average velocity of a mouse. (**B**). Distance moved by the animals in the central region of the arena. * $-p < 0.05$; *** $-p < 0.001$. Solid triangles, circles and squares depict control IgG, naïve and SBI-IgG injected mice, respectively.

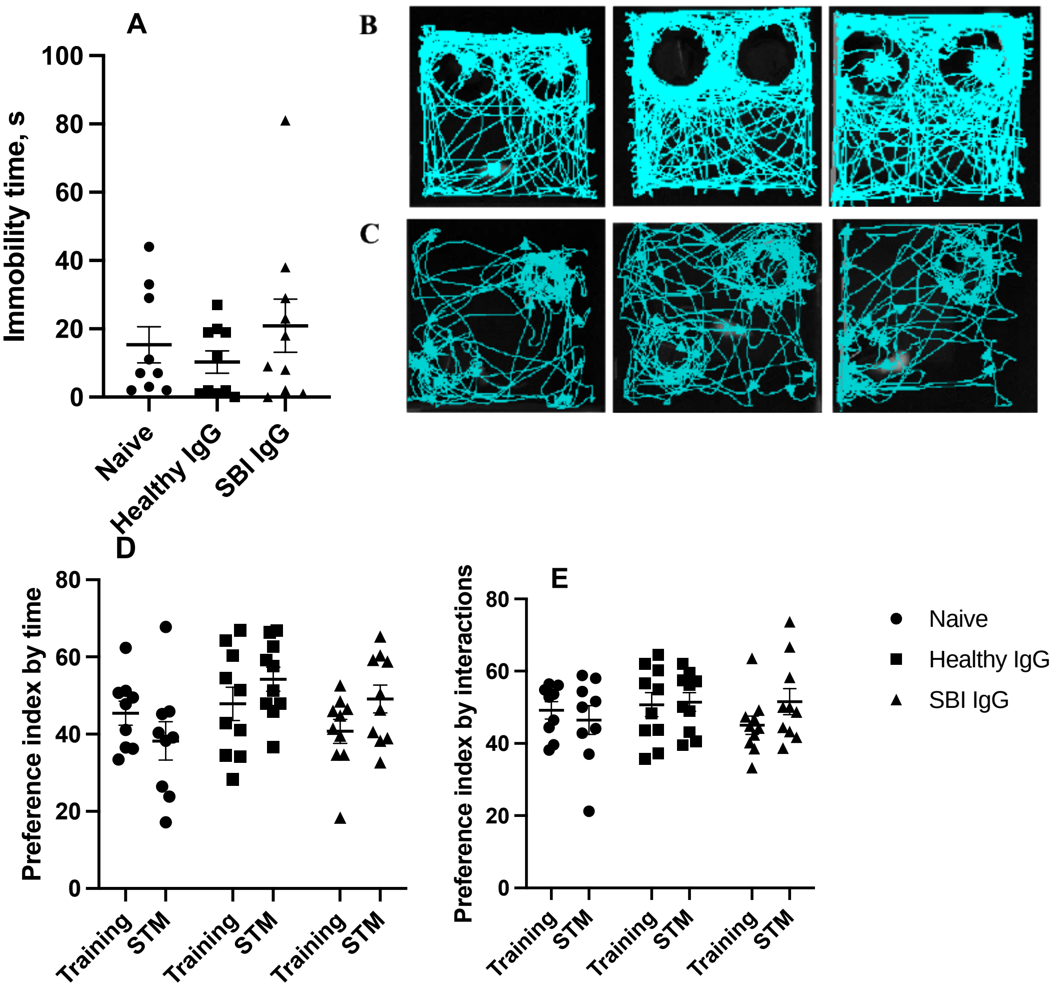

Supplementary Figure S3: Forced swim test and short-term version of novel object location paradigm. (**A**). Forced swim test conducted on the experimental groups. Immobility time measured during the last 2 minutes of the paradigm (6 min). (**B**)–(**E**). Novel object location test. (**B**) and (**C**). Representative trajectories of mice from each group per training (**B**) and learning (**C**) phases. Location index as defined in Methods derived from time spent near (**D**) and interaction with (**E**) the novel location of the object. Data presentation as in Figure 4.
